# New p35 (H3L) Epitope Involved in Vaccinia Virus Neutralization and Its Deimmunization

**DOI:** 10.3390/v14061224

**Published:** 2022-06-05

**Authors:** Yana Khlusevich, Andrey Matveev, Lyudmila Emelyanova, Elena Goncharova, Natalia Golosova, Ivan Pereverzev, Nina Tikunova

**Affiliations:** 1 Laboratory of Molecular Microbiology, Institute of Chemical Biology and Fundamental Medicine, Siberian Branch of Russian Academy of Sciences, 630090 Novosibirsk, Russia; guterus@gmail.com (A.M.); mila.kuharenko@mail.ru (L.E.); n.golosova@g.nsu.ru (N.G.); 2 Laboratory of Nucleic Acids Biochemistry, Institute of Chemical Biology and Fundamental Medicine, Siberian Branch of Russian Academy of Sciences, 630090 Novosibirsk, Russia; egn@niboch.nsc.ru; 3 Laboratory of Synthetic Biology, Institute of Chemical Biology and Fundamental Medicine, Siberian Branch of Russian Academy of Sciences, 630090 Novosibirsk, Russia; pereverzevi1994@mail.ru

**Keywords:** H3L, neutralizing antibodies, vaccinia virus, immunogenicity, deimmunization, epitope, oncolytic virus, oncolytic vaccinia virus, p35, orthopoxvirus

## Abstract

Vaccinia virus (VACV) is a promising oncolytic agent because it exhibits many characteristic features of an oncolytic virus. However, its effectiveness is limited by the strong antiviral immune response induced by this virus. One possible approach to overcome this limitation is to develop deimmunized recombinant VACV. It is known that VACV p35 is a major protein for B- and T-cell immune response. Despite the relevance of p35, its epitope structure remains insufficiently studied. To determine neutralizing epitopes, a panel of recombinant p35 variants was designed, expressed, and used for mice immunization. Plaque-reduction neutralization tests demonstrated that VACV was only neutralized by sera from mice that were immunized with variants containing both N- and C- terminal regions of p35. This result was confirmed by the depletion of anti-p35 mice sera with recombinant p35 variants. At least nine amino acid residues affecting the immunogenic profile of p35 were identified. Substitutions of seven residues led to disruption of B-cell epitopes, whereas substitutions of two residues resulted in the recognition of the mutant p35 solely by non-neutralizing antibodies.

## 1. Introduction

The *Orthopoxvirus* is the most well-known genus of the Poxviridae family, which contains complex DNA viruses. All viruses of this genus replicate in the cytoplasm of infected cells, because they have their own replication and transcription regulatory mechanisms [1,2]. Some orthopoxviruses are pathogenic for humans, e.g., variola virus (VARV) is a causative agent of smallpox, monkeypox virus (MPXV) causes zoonotic exanthemata disease [3,4,5,6,7], whereas infection with vaccinia virus (VACV) or cowpox virus (CPXV) usually leads to local skin damage [1]. Although smallpox was eradicated, orthopoxviruses continue to be a source of biological danger to humans because orthopoxviruses still circulate in nature and are capable of infecting humans. MPXV can cause both sporadic cases of human smallpox-like disease and outbreaks of this infection [8,9,10]. In addition, human cases of cowpox and vaccinia-like diseases are constantly being recorded [11,12,13,14,15,16,17,18,19,20,21]. Vaccination with live VACV leads to the formation of long-term immunity against orthopoxviruses in vaccinated people [22]. However, as a result of the elimination of smallpox, mass vaccination was discontinued in the second half of the 1970s and the majority of the population currently has no immunity against orthopoxvirus infections. Vaccination with live VACV is sometimes accompanied by serious postvaccination complications [23,24]. In this regard, the development of new vaccines based on attenuated VACV and therapeutics for the treatment of infections caused by orthopoxviruses does not stop [25].

In addition, VACV is considered a promising oncolytic agent, because VACV has many characteristics that make it an ideal platform for developing oncolytic agents. VACV has a short life cycle that takes place in the cytoplasm, which eliminates the risk of genome integration, and demonstrates notable benefits, such as efficient delivery, large transgene-encoding capacity, intravenous stability, and the ability to induce efficient immune responses [26,27]. Moreover, even a single application of an oncolytic VACV can form antiviral immunity, including virus neutralizing antibodies [27]. If a person has already been vaccinated with VACV in childhood or received this vaccine to prevent infection with MPXV, the first administration of the oncolytic VACV would probably be useless due to pre-existing immunity. 

In order to avoid a strong antiviral immune response, a VACV deimmunization strategy is required. One possible approach to deimmunization is to mask or eliminate the most effective neutralizing epitopes of the virus. This approach has been successful in developing antibody-escaping variants of the measles virus [28,29], influenza virus [30], hepatitis B and C virus [31], human immunodeficiency virus [32,33], adenovirus, and adeno-associated vectors (AAVs) [34,35].

Among several dozens of VACV proteins found in the sera of vaccinated individuals, p35 encoded by the H3L open reading frame (ORF) is one of the major immunogenic proteins [36,37,38,39,40,41,42]. The VACV p35 protein induces both T-cell and B-cell immune reactions and this protein exposes at least two confirmed T-cells epitopes [43,44]. Anti-p35 antibodies are detected in most sera of VACV-immunized persons [45]. This protein is a major target for neutralizing antibodies in humans and can protect mice against lethal challenge with VACV [40,41,42,46]. Despite the relevance of orthopoxvirus p35, its epitope structure remains insufficiently studied. Using CPXV-derived recombinant p35 proteins, the p35 region bound by a neutralizing human monoclonal antibody was previously determined [46]. In addition, the peptide phage display approach indicated that this epitope is discontinuous and probably localized on p35 loops 13–34 amino acid residues (aa) and 231–239 aa. In this study, the VACV p35 region that was recognized by the anti-VACV polyclonal neutralizing antibodies was located. In addition, several aa for the neutralizing epitope deletion strategy were identified using a random mutagenesis strategy.

## 2. Materials and Methods

### 2.1. Cells, Sera, and Viruses 

CV-1 cells were obtained from the cell culture repository of the Institute of Chemical Biology and Fundamental Medicine, Siberian Branch of the Russian Academy of Sciences. Cells were grown in Iscove’s Modified Dulbecco’s Medium (IMDM, Thermofischer Scientific, Waltham, MA, USA) containing 10% heat inactivated fetal bovine serum (FBS, Thermofischer Scientific, Waltham, MA, USA) and antimicotics-antibiotics solution (100 U/mL of penicillin and 0.1 mg/mL of streptomycin, Thermofischer Scientific, Waltham, MA, USA) in an atmosphere of 5% CO_2_ at 37 °C.

Attenuated vaccinia virus (VV) LIVP/GFP (TK-) strain containing an insertion of the green fluorescent protein (GFP) gene in the viral thymidine kinase gene was grown and titrated on CV-1 cells, as described previously [47].

The sera from vaccinated donors were obtained as part of the research performed previously [41].

### 2.2. Production of Recombinant VACV p35 Proteins

All recombinant p35 fragments were derived from H3L ORF, VACV strain LIVP (GenBank KP233807.1) and produced as fusion proteins with thioredoxin and a C-terminal His tag. Gene fragments p35Δ (1–282 aa), p35Δ3 (1–135 aa), p35Δ6 (128–282 aa), and p35Δ12 (1–239 aa) were obtained using a set of primers (Table 1). In addition, an artificial epitope p35Δfuse was constructed from two gene fragments (1–34 aa and 228–239 aa) joined by a flexible peptide linker (GGGS)_3_. To develop the p35Δfuse, two overlapping primers p35fuseN and p35fuseC and extension PCR were used. The resulting PCR product containing the p35Δfuse was obtained using primers H3L Bam-1+ and H3L Back12-EcoRI. All obtained PCR fragments were digested with *Bam*HI and *Eco*RI and ligated into BamHI/PstI-digested expression plasmid pET32a (Novagen, Madison, WI, USA). *Escherichia coli* BL21(DE3) cells were transformed with the resulting plasmids pET32-p35Δ, pET32-p35Δ3, pET32-p35Δ6, pET32-p35Δ12, and pET32-p35Δfuse and plated independently onto agar with 50 mkg/mL ampicillin and incubated at 37 °C overnight. *E. coli* clones were screened for the presence of the appropriate gene fragment by PCR using primers pET32a-dir (5′-TGCTAGTTATTGCTCAGCGG-3′) and pET32a-rev (5′-GGTTCTGGTTCTGGCCATA-3′), followed by sequencing. 

In each case, a confirmed positive colony was grown in 100 mL of LB medium containing 50 μg/mL of ampicillin at an agitation rate of 180 rpm at 37 °C. When the optical density OD_600_ reached 0.6–0.8, protein synthesis was induced by adding 0.1 mM of isopropyl β-D-1-thiogalactropyranoside (IPTG). Then, cells were cultured for 4 h under the same conditions. After that, the cells were centrifuged for 10 min at 6000× *g* and resuspended in a phosphate buffered saline (PBS), containing 1 mM PMSF, pH 7.5. The cell pellet was homogenized using an ultrasonic homogenizer Sonopuls HD 2070 (Bandelin, Berlin, Germany) for 10 min at 35% amplitude, with 10 s of sonication followed by a 10 s rest period. Subsequently, the samples were centrifuged at 15,000× *g* for 20 min. The obtained soluble cytoplasm fractions were used for proteins purifications.

Recombinant proteins rp35Δ, rp35Δ3, rp35Δ6, rp35Δ12, and rp35Δfuse were purified from cytoplasm using Ni-NTA agarose (Qiagen, Hilden, Germany). For chromatography, PBS was used with the addition of NaCl up to 300 mM (wash buffer). Nonspecific proteins bound with Ni-NTA were eluted with a wash buffer, containing 50 mM imidazole, after which the truncated p35 protein was eluted with a buffer containing 250 mM imidazole. After purification, protein dialysis was performed using a storage buffer, containing 50 mM Tris-HCl, 150 mM NaCl, pH 7.5. Proteins were concentrated to 1–2 mg/mL in storage buffer, using centrifuge concentrator «Vivaspin turbo 15 PES» with a 10 kDa cutoff (Sartorius, Germany).

### 2.3. Mice Immunization

Three-month-old male BALB/c mice were obtained from the animal care facility in ICBFM, Novosibirsk. Animals were housed under a normal light–dark cycle; water and food were provided ad libitum. All animal procedures were carried out in accordance with the recommendations for the protection of animals used for scientific purposes (EU Directive 2010/63/EU). All experiments with animals were approved by the Inter-institutional Bioethics Committee of Institute of Cytology and Genetics Siberian Branch of Russian Academy of Sciences, Novosibirsk, Russia.

Mice were immunized intraperitoneally (i.p.) with 50 μg of truncated proteins per mouse, three times with an interval of 14 days between immunizations. Incomplete Freund’s adjuvant was used for the first immunization, whereas incomplete Freund’s adjuvant was used for the second and third immunizations. Each experimental group of mice included 10 animals; mice from the control group were immunized with 0.9% NaCl. Blood samples were taken from the facial vein 2 week after the final immunization.

### 2.4. Western Blot Analysis 

VACV or recombinant proteins were resolved by 12.5% SDS-polyacrylamide gel electrophoresis and transferred onto a nitrocellulose membrane (Bio-Rad Laboratories, Hercules, CA, USA). After blocking with 5% dry skim milk, the membrane was incubated with a 1\10 dilution of human or mice pooled sera in PBS, containing 0.1% Tween 20% for 1 h at 37 °C. Then, the membrane was incubated with alkaline phosphatase-conjugated anti-human IgG rabbit antibodies (Thermofischer Scientific, Waltham, MA, USA) or alkaline phosphatase-conjugated anti-mouse IgG rabbies antibodies (Thermofischer Scientific, Waltham, MA, USA). Protein–antibody interactions were visualized by incubating the membrane in a mixture of nitro blue tetrazolium (NBT, VWR, Radnor, PA, USA) and 5-bromo-4chloro-3-indolyl-phosphate (BCIP, Roche, Basel, Switzerland) for 20 min at room temperature. The serum of a volunteer who had never been vaccinated nor infected by orthopoxviruses and normal mouse serum were used as corresponding negative controls.

### 2.5. Enzyme-Linked Immunosorbent Assay (ELISA)

For indirect ELISA, the wells of 96-well microtiter plate were independently coated with 200 ng of truncated VACV p35 proteins per well or a control antigen in PBS, pH 7.4. In the study, the trx-NS1 protein of the tick-borne encephalitis virus [48], produced and purified as described in Section 2.2, was used as a control antigen. After blocking with 3% bovine serum albumin in PBS, 10-fold dilutions of vaccinated human sera or immunized mice sera were added to each well; the starting dilution was 1:2 for human sera and 1:10 for mice sera. Indirect ELISA was conducted with alkaline phosphatase-conjugated anti-human IgG rabbit antibodies (Thermofischer Scientific, Waltham, MA, USA) or alkaline phosphatase-conjugated anti-mouse IgG rabbit antibodies (Thermofischer Scientific, Waltham, MA, USA) and stained with p-nitrophenyl phosphate. Absorbance was measured at 405 nm using a microtiter reader iMark (Bio-Rad Laboratories, Hercules, CA, USA). The blood serum of a volunteer who had never been vaccinated nor infected by orthopoxviruses was used and normal mouse serum was used as corresponding controls of nonspecific binding.

### 2.6. Antibody Depletion

Depletion of specific antibodies from anti-Trx-p35Δ mice sera was performed using Dynabeads m270 Epoxy (Thermofischer Scientific, Waltham, MA, USA) according to the manufacturer manual. For this purpose, 100 μg rp35Δ, rp35Δ3, rp35Δ6, rp35Δ12, or rp35Δfuse was covalent bound to 5 μg paramagnetic beads for 20 h at 37 °C in phosphate buffer pH 7.5 with slow tilt rotation, and after a washing step with PBS, 500 μL of 5-fold diluted serum was added and incubated for 3 h at 37 °C. Thereafter, magnetic beads were pelleted by magnetic force and depleted sera were obtained. Bound antibodies were eluted from beads using 0.1 M citrate buffer pH 3.1. Three rounds of antibody depletion were performed similarly. The absence of specific antibodies was confirmed by ELISA with the antigen being used for depletion.

### 2.7. Plaque-Reduction Neutralization Test (PRNT)

VACV suspension diluted in IMDM and supplemented with 10% FBS was mixed with an equal volume of serial two-fold dilutions of sera or depleted sera (starting serum dilutions were 1:10). The mixtures were incubated at 24 °C for 2 h and then added to confluent CV-1 cell monolayers in 24-well culture plates (TPP, Switzerland) and incubated for 1 h at 37 °C. Then, the mixtures were removed and the cells were washed with IMDM and overlaid with IMDM containing 10% FBS. Two days after infection, the viable cells were stained with 2% crystal violet with 10% formalin, and clear plaques were visualized. The normal mouse serum was used as a control of nonspecific neutralization. Neutralization titer was calculated according to N = (V_0_ − Vn)/V_0_ × 100%, where V_0_ is the number of plaques in control wells without serum, and Vn is the number of plaques in tested wells.

### 2.8. Random Mutagenesis of rp35Δfuse 

Mutant variants of rp35fuse protein were obtained using H3L Bam-1+ and H3L Back12-EcoRI primers and PickMutant™ Error Prone PCR Kit (Canvax, Spain) according to the manufacturer’s recommendations. Mutated PCR fragments were inserted into the expression plasmid pET32a and sequenced. Mutant rp35Δfuse proteins were obtained as described in Section 2.2.

### 2.9. Statistical Analyses

Data were analyzed with GraphPad Prism software, version 7. Two-tailed t tests were used to compare antibody titers in sera and PRNT_50_. Differences were considered significant when the *p* value was less than 0.05. 

## 3. Results

### 3.1. Characterization of Recombinant p35 Antigens

**Figure 1:**
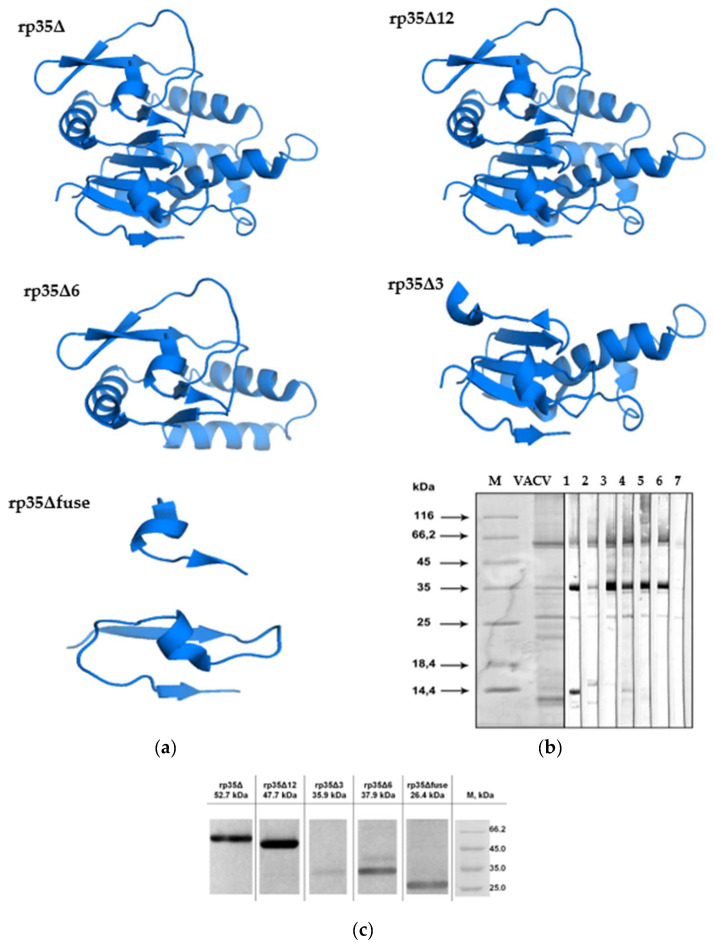
Characterization of truncated variants of VACV p35: (**a**) Ribbon and surface representations of rp35Δ, rp35Δ12, rp35Δ6, rp35Δ3, rp35Δfuse, predicted using a homology-based online modeling service I-TASSER (http://zhanglab.ccmb.med.umich.edu/I-TASSER/, access date 14 April 2022) and molecular coordinates for the VACV p35 protein (PDB 5EJ0); (**b**) Western blot analysis of VACV proteins fractionated by SDS-PAGE (12%) and developed with serum samples obtained from immunized (lanes1–6) and a nonimmunized (lane 7) volunteers, M, protein molecular weight markers; (**c**) Western blot analysis of purified recombinant variants of VACV p35, which were fractionated by SDS-PAGE (12.5%) and developed with anti-VACV pooled human sera diluted to 1:200.

Several recombinant VACV-derived p35 antigens for immunoassay, neutralization, and depletion analysis were produced and purified as described in Section 2.2. These recombinant p35 fragments were designed using a homology-based online 3D modeling service I-TASSER (http://zhanglab.ccmb.med.umich.edu/I-TASSER/, access date 14 April 2022) and molecular coordinates for VACV p35 H3 protein (PDB 5EJ0) obtained from the Protein Data Bank [36]. More specifically (Figure 1a), rp35Δ (1–282 aa) corresponds to p35 of VACV without the C-terminal transmembrane region, rp35Δ3 (1–135 aa) and rp35Δ6 (128–282 aa) contain N- and C-terminal parts of the p35Δ, respectively, and p35Δ12 (1–239 aa) includes a region, with localized orthopoxvirus neutralizing epitopes according to a previous study [46]. In addition, the recombinant epitope p35Δfuse was developed based on the neutralizing epitope predicted by the peptide phage display [46]. The Trx-tag fusion partner was added to each p35 variant to provide correct folding and to increase the solubility of the recombinant proteins in *E. coli*.

To confirm that all recombinant p35 proteins contain epitopes that are recognized by human polyclonal antibodies, the proteins were subjected to Western blot analysis with human sera from VACV-LIVP-vaccinated volunteers. Before the experiment, anti-VACV polyclonal antibodies from vaccinated volunteers were individually tested by Western blot analysis for their binding with VACV-LIVP proteins. Six serum samples that had exhibited the most effective binding (Figure 1b) were selected and pooled for Western blot analysis. Expectedly, pooled anti-VACV human policlonal antibodies recognized rp35Δ and rp35Δ12, which contain the most immunogenic regions of orthopoxvirus p35. Among truncated recombinant p35 variants, rp35Δ6 and rp35Δfuse, but not rp35Δ3, were revealed in pooled human sera (Figure 1c). The obtained results demonstrated that Trx-p35Δ6 and Trx-p35Δfuse expose B-specific epitopes recognized by human anti-VACV polyclonal antibodies and these epitopes are probably localized in regions 1–34 aa and 128–282 aa. 

### 3.2. Mice Immunization with Recombinant VACV p35 Proteins

**Figure 2:**
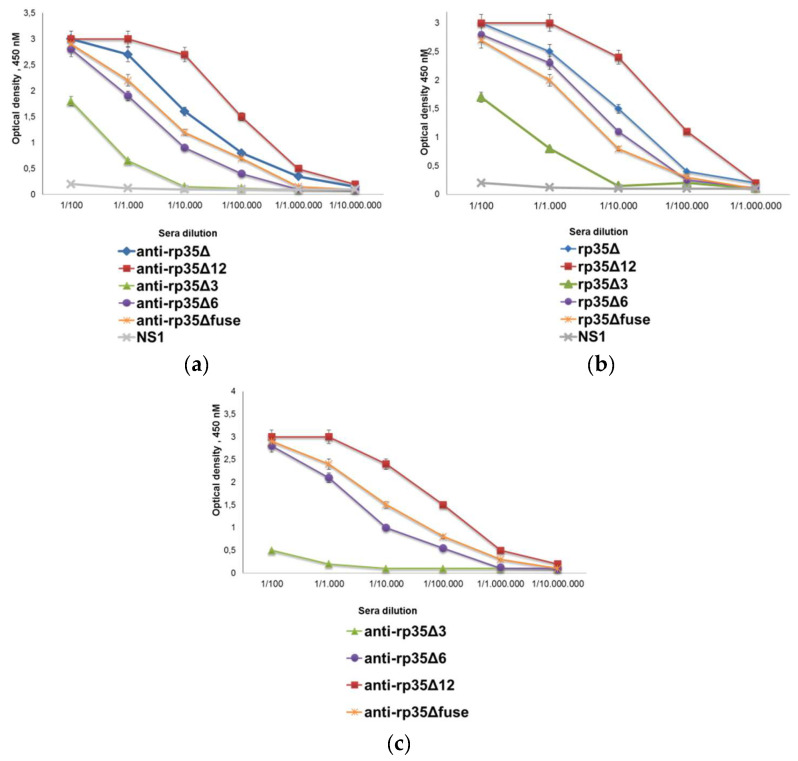
Reactivities of obtained sera samples: (**a**) Indirect ELISA evaluating binding of pooled anti-rp35Δ, anti-rp35Δ3, anti-rp35Δ6, anti-rp35Δ12, and anti-rp35Δfuse sera with the corresponding immunogen; (**b**) indirect ELISA evaluating binding of pooled anti-rp35Δ sera with rp35Δ, rp35Δ3, rp35Δ6, rp35Δ12, and rp35Δfuse; (**c**) indirect ELISA evaluating binding of pooled anti-rp35Δ3, anti-rp35Δ6, anti-rp35Δ12, and anti-rp35Δfuse with rp35Δ. Tick-borne encephalitis trx-NS1 was used as a control of nonspecific binding. Absorbance was measured at 450 nm.

Five groups of ten BALB/c mice were immunized with purified rp35Δ, rp35Δ3, rp35Δ6, rp35Δ12, and rp35Δfuse. In each group, serum samples were taken 2 weeks after the final immunization and pooled, resulting in five pooled sera. The antibody response induced by rp35Δ rp35Δ3, rp35Δ6, rp35Δ12, and rp35Δfuse was tested in indirect ELISA (Figure 2a). 

Pooled serum samples obtained from mice immunized with rp35Δ, rp35Δ6, rp35Δ12, and rp35Δfuse demonstrated positive signals in binding with the corresponding immunogen, diluted to 1:10,000 or more. The antibody response induced by rp35Δ3 was lower and anti-rp35Δ3 polyclonal antibodies revealed this antigen to be diluted to 1:1000 (Figure 2a). In addition, serial dilutions of pooled anti-rp35Δ polyclonal antibodies were tested for binding with rp35Δ3, rp35Δ6, rp35Δ12, and rp35Δfuse. The results demonstrated that anti-rp35Δ mice polyclonal antibodies recognized all tested recombinant p35 variants, including an artificial epitope rp35Δfuse (Figure 2b). In turn, polyclonal antibodies from pooled serum samples obtained from mice immunized with rp35Δ6, rp35Δ12, and rp35Δfuse bound both the corresponding immunogen and rp35Δ with high titer (Figure 2c). An exception was the anti-rp35Δ3 pooled serum sample, which recognized rp35Δ to a far lesser extent than rp35Δ3 (Figure 2).

**Figure 3:**
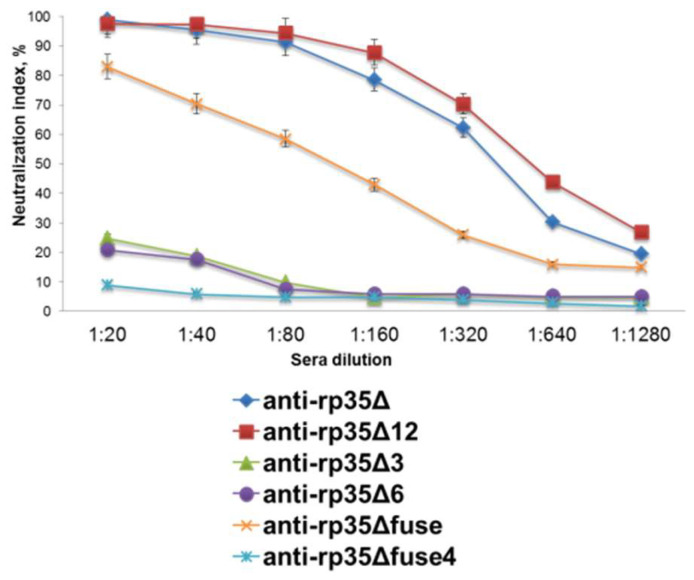
In vitro neutralization of VACV with serial dilutions of pooled anti-rp35Δ, anti-rp35Δ3, anti-rp35Δ6, anti-rp35Δ12, anti-rp35Δfuse, and anti-rp35Δfuse4 sera samples. The initial dilution of sera was 1:20. VACV was diluted to 320 pfu/mL. The data from three or more independent experiments are shown.

To study the contribution of various epitopes in eliciting neutralizing antibodies, pooled serum samples obtained from mice immunized with rp35Δ, rp35Δ3, rp35Δ6, rp35Δ12, and rp35Δfuse were tested for their ability to neutralize VACV in PRNT. It was shown that anti-rp35Δ and anti-rp35Δ12 mice polyclonal antibodies reduced the number of plaques by more than 50% (PRNT50) after being diluted to 1:320, whereas anti-rp35Δfuse antibodies neutralized VACV at a dilution of 1:160 (Figure 3). Anti-rp35Δ3 and anti-rp35Δ6 pooled sera demonstrated weak neutralizing activity and, diluted to 1:20, did not reduce the number of plaques by 50% (Figure 3). The obtained results indicated that both the 1–135 aa and 128–282 aa regions of VACV p35 contain epitopes eliciting non-neutralizing or weakly neutralizing antibodies. 

### 3.3. Depletion Analysis

**Figure 4:**
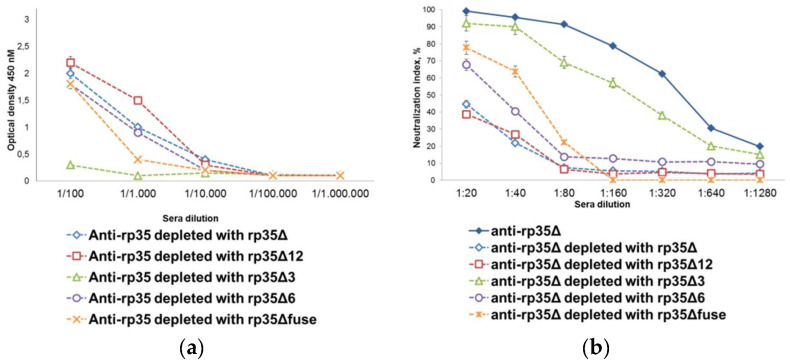
Characterization of pooled anti-rp35Δ sera after depletion. (**a**) Indirect ELISA evaluating binding of depleted anti-rp35Δ sera with the corresponding depletion antigen; (**b**) in vitro neutralization of VACV with serial dilutions of pooled anti-rp35Δ sera depleted with rp35Δ, rp35Δ3, rp35Δ6, rp35Δ12, and rp35Δfuse. The initial dilution of sera was 1:20. VACV was diluted to 320 pfu/mL. The data from three or more independent experiments are shown.

The pooled anti-rp35Δ mice serum sample was depleted with rp35Δ, rp35Δ3, rp35Δ6, rp35Δ12, and rp35Δfuse. Depletion was controlled by ELISA with corresponding depletion antigens; depletion was considered complete if the ELISA threshold was less than 5% residual reactivity (Figure 4a).

The effect of the removal of distinct antibody subsets was then determined in PRNT. Pooled anti-rp35Δ mice sera after depletion with rp35Δ or rp35Δ12 almost completely lost their neutralizing activity and did not reduce the number of VACV plaques by more than 50% even after being diluted to 1:20 (Figure 4b). Depletion with rp35Δ6 and rp35fuse also resulted in a reduction in neutralizing activity, although to a lesser extent; depleted anti-rp35Δ mice sera reduced the number of VACV plaques by more than 50% at dilutions of 1:20 and 1:40, respectively. Notably, rp35Δ3 almost did not reduce the neutralizing activity of the pooled anti-rp35Δ mice sera (Figure 4b). The obtained results confirmed that neutralizing epitopes are displayed completely or at least to a large extent in the 1–239 aa region of VACV p35. 

### 3.4. rp35Δfuse Random Mutagenesis 

**Figure 5:**
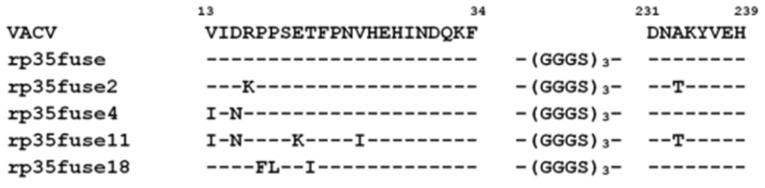
Alignment of rp35Δfuse mutant variants.

Since rp35Δfuse is the shortest of the obtained truncated p35 variants (46 aa) that elicit anti-VACV neutralizing antibodies, a panel of rp35Δfuse mutants was developed using error-prone PCR. The obtained PCR-fragments were inserted into a pET32a plasmid and their nucleotide sequences were determined. Four unique mutant genes were identified (Figure 5). Mutant proteins rp35Δfuse2 (R16K, A233T), rp35Δfuse4 (V13I and D15N), rp35Δfuse11 (V13I, D15N, E20K, V25I, A233T), and rp35Δfuse18 (P17F, P18L, T21I) were produced in *E. coli* BL21(DE3) cells and purified as described previously.

**Figure 6:**
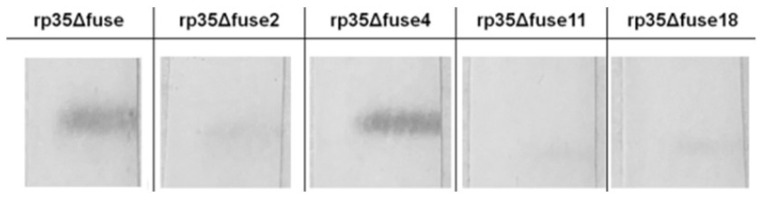
Characterization of mutated variants of rp35Δfuse. Western blot analysis of purified mutated variants of rp35Δfuse, which were fractionated by SDS-PAGE (12.5%) and developed with neutralizing pooled human sera of volunteers vaccinated with VACV (diluted to 1:200).

Purified rp35Δfuse2, rp35Δfuse4, rp35Δfuse11, and rp35Δfuse18 proteins were subjected to Western blot analysis with anti-VACV polyclonal human antibodies from the pooled sera of VACV-vaccinated volunteers. Only rp35Δfuse4 was bound by the specific anti-VACV polyclonal human antibodies similar to rp35Δfuse, whereas rp35Δfuse2, rp35Δfuse11, and rp35Δfuse18 were not recognized by them (Figure 6). The obtained data demonstrated that aa R16, P17, P18, E20, T21, V25, or A233 could be involved in forming B-cell epitopes and their substitution led to masking these epitopes.

The mutant protein rp35Δfuse4 recognized by the sera of VACV-vaccinated volunteers was used for mice immunization, as was described in Section 2.3. The obtained anti-rp35Δfuse4 serum samples were pooled and tested for neutralizing activity against VACV in PRNT. No neutralizing activity was found (Figure 3). These data indicated that substitution V13I or D15N, or both of them, changed a neutralizing epitope on p35 to an epitope recognized by non-neutralizing human antibodies.

## 4. Discussion

VACV is a promising candidate for the development of new cancer therapies. However, its effectiveness is limited by the strong antiviral immune response induced by VACV. One possible approach to overcome this limitation is to develop low-immunogenic recombinant VACV by masking epitopes that elicit neutralizing antibodies. Here, this approach was used for the deimmunization of VACV p35, which is a major target for neutralizing antibodies in humans.

First, the p35 region of VACV that exposes most neutralizing epitopes was determined. It was confirmed that most, or even all, neutralizing epitopes are localized between 1 and 239 aa, and they are exposed on rp35Δ12 (1–239 aa) for neutralizing polyclonal antibodies from sera similar to, or even better than, on rp35Δ (1–282 aa). Unexpectedly, the N- and C-terminal parts of rp35Δ, namely, rp35Δ3 (1–135 aa) and rp35Δ6 (129–282 aa), did not elicit strong neutralizing antibodies when being used for mice immunization. In addition, they did not deplete anti-rp35Δ neutralizing polyclonal antibodies. Considering that 3D modeling showed that rp35Δ3 and rp35Δ6 maintained their structures similar to the corresponding parts of rp35Δ (Figure 1A), we concluded that the neutralizing epitope or epitopes are discontinuous. This suggestion was confirmed when the immune properties of rp35Δfuse (1–34 – (GGGS)_3_–228–239 aa) were examined. This artificial antigen elicited neutralizing antibodies in mice and bound neutralizing antibodies in anti-rp35Δ sera in depletion experiments. These characteristics in rp35Δfuse were slightly reduced as compared with these characteristics in rp35Δ and rp35Δ12, which can be explained by the structure of rp35Δfuse, which did not fully correspond to the conformation of the appropriate regions in rp35Δ. Previously, a discontinuous epitope recognized by a neutralizing human monoclonal antibody was localized on the p35 of CPXV within two p35 regions, i.e., 15–19 aa and 232–237 aa [46]. Our results are in good agreement with previously published results.

Second, we determined which aa are involved in forming the epitope recognized by anti-VACV neutralizing antibodies. The obtained data demonstrated that aa R16, P17, P18, E20, T21, V25, and A233 are crucial and their substitutions led to the formation of epitopes unrecognizable by human anti-VACV polyclonal antibodies. The aa necessary for binding with polyclonal antibodies are localized on loops ^13^VIDRLPSETFPNVHEHINDQKF^34^ and ^231^DNAAKYVEH^239^, which are available for antibodies, as shown in the 3D structure of the VACV p35 fragment [36]. We assume that substitutions of P17, P18, T21, E20, V25, and A233 result in incorrect folding of mutant p35 and a disruption of the discontinuous epitope recognized by human antibodies. 

Two other substitutions, V13I and D15N, affected B-cell immunity in a more complicated way. They did not affect the recognition of the epitope containing them by anti-VACV human polyclonal antibodies; however, the mutant protein with these substitutions only elicited non-neutralizing antibodies in mice. Thus, some p35 substitutions can eliminate B-cell epitopes on the protein, whereas other substitutions can only mask epitopes from neutralizing antibodies.

Our data, which were obtained by random mutagenesis, are partly consistent with those from a previous study of linear p35 epitopes, which was performed using alanine scanning investigation of epitopes [49]. In this study, 21 aa of the VACV p35 involved in recognition by human antibodies were identified [49]. Among them, aa I14, D15, and R16 were confirmed by both approaches. Importantly, when substitutions are introduced into p35, VACV remains viable. It was previously shown that the deletion of ORF H3L leads to a decrease in the infectivity of VACV and in the size of viral plaques in cell culture [42]. This protein has a dual function in the life cycle of orthopoxviruses: it is involved in the attachment of the virion to the cell and in the process of viral morphogenesis [50]. Substitutions found in our study were not localized in the glycosaminoglycan-binding sites of p35 (94–101 aa and 159–164 aa), which are responsible for binding with heparan sulfates exposed on the surface of many eukaryotic cells. Therefore, these substitutions could not affect the attachment of VACV to the cancer cell. In addition to p35 (H3L), glycoproteins L1R, A27L, and D8L were identified as major immunogenic proteins [36,37,51,52,53,54,55,56]. For L1R, a single amino acid substitution was detected, which makes VACV completely resistant to neutralization by antibodies [57]. Substitutions and deletions in A27L and D8L for deimmunization are also possible, since A27L and D8L knockout VACV remains viable, although its infectivity decreases [58,59].

In conclusion, an epitope that elicits strong neutralizing antibodies in humans was localized on the VACV p35 surface. At least nine aa in p35 were shown to be crucial. Substitutions of seven of them prevent binding of anti-VACV human polyclonal antibodies with mutant p35, whereas substitutions of two aa result in the recognition of the mutant p35 solely by non-neutralizing antibodies. The identified aa could be useful for the development of low-immunogenic recombinant VACV.

## Figures and Tables

**Table 1:** Primers used for the development of recombinant p35 of VACV.

Truncated p35	Primer Name	Primer Sequences	Amino Acids
p35Δ	H3L Bam-1+	5′-GCGCGGGATCCGGTGGAATGGCGGCGGTGAAAAC-3′	1–282
	H3L Back-EcoRI	5′-GGCTTGAATTCCCAAATGAAATCAGTGGAGTAGT-3′	
p35Δ3	H3L Bam-1+	5′-GCGCGGGATCCGGTGGAATGGCGGCGGTGAAAAC-3′	1–135
	H3L Back3-EcoRI	5′-GGCTTGAATTCCCAAACGTAATATCCTCAATAAC-3′	
p35Δ6	H3L Bam-6+	5′-GCGCGGGATCCGTTATTGAGGATATTACGTTTC-3′	128–282
	H3L Back-EcoRI	5′-GGCTTGAATTCCCAAATGAAATCAGTGGAGTAGT-3′	
p35Δ12	H3L Bam-1+	5′-GCGCGGGATCCGGTGGAATGGCGGCGGTGAAAAC-3′	1–239
	H3L Back12-EcoRI	5′-GGCTTGAATTCCCGTGTTCTACATATTTGGCGGCG-3′	
p35ΔFuse	p35fuseN	5′-GCGCGGGATCCGGTGGAATGGCGGCGGTGAAAACTCCTGTTATTGTTGTGCCAGTTATTGATAGACCCCCATCAGAAACATTTCCTAATGTTCATGAGCATATTAATGATCAGAAG-3′	1–34–(GGGS)_3_–228–239
	p35fuseC	5′-CTTGGCTGCAGGTGTTCTACATATTTGGCGGCGTTATCCAGTATCTGCGACCCTCCACCAGAA CCTCCGCCTGAACCGCCTCCGCTGAACTTCTGATCATTAATATGCTCATGA-3′	

## Data Availability

Not applicable.
